# 8-Oxoguanine transcriptional miscoding can transiently reproduce PAH-associated premature-stop transcripts

**DOI:** 10.64898/2026.10.01.756055

**Published:** 2026-10-07

**Authors:** Santiago Moreno-Caceres, Alexis M. Hayes, Amber Delmas Eliason, David P. Marciano

## Abstract

Pulmonary arterial hypertension (PAH) is associated with increased oxidative DNA damage, while haploinsufficiency of BMPR2 and other dosage-sensitive genes contributes directly to disease. Template-strand 8-oxoguanine (8-oxoG) provides a potential transient route to the same loss-of-function outcome through transcriptional mutagenesis without altering genomic DNA. RNA polymerase II can insert adenine opposite the lesion and generate a coding-sense C>A error in the nascent transcript. We asked whether this chemistry could generate premature-stop transcripts in dosage-sensitive PAH genes. Across 17,880 C>A-accessible coding positions in pulmonary vascular genes, 1,192 could generate premature termination codons, including 883 predicted to engage nonsense-mediated decay (NMD). Restricting these sites to expressed autosomal-dominant PAH genes with definitive ClinGen validity and established haploinsufficiency identified 116 NMD-prone positions in BMPR2, KDR, SMAD9, and TBX4. Eight exactly reproduce ClinVar pathogenic or likely pathogenic stop variants, including five in BMPR2. At BMPR2 c.1424, template-strand 8-oxoG and the expert-panel-reviewed genomic c.1424C>A allele is predicted to generate the same UAA-containing transcript. Published oxidized-guanine maps detect signal at seven of the eight coordinates, providing coordinate-level support for oxidative modification at these sites. These findings define a sequence-resolved route through which repairable oxidative DNA damage could transiently reproduce loss-of-function transcripts encoded by pathogenic PAH alleles without permanent genomic mutation.

## Full Text Availability

The license terms selected by the author(s) for this preprint version do not permit archiving in PMC. The full text is available from the preprint server.

